# Oral pigmentation as an adverse effect of chloroquine and hydroxychloroquine use: A scoping review

**DOI:** 10.1097/MD.0000000000029044

**Published:** 2022-03-18

**Authors:** Pedro Urquiza Jayme Silva, Millena Barroso Oliveira, Walbert Vieira, Sérgio Vitorino Cardoso, Cauane Blumenberg, Ademir Franco, Walter Luiz Siqueira, Luiz Renato Paranhos

**Affiliations:** ^a^ *Postgraduate Program in Dentistry, School of Dentistry, Federal University of Uberlandia, Uberlândia, Minas Gerais, Brazil,* ^b^ *Department of Restorative Dentistry, Endodontics Division, School of Dentistry of Piracicaba, State University of Campinas, UNICAMP, Piracicaba, São Paulo, Brazil,* ^c^ *Division of Pathology, School of Dentistry, Federal University of Uberlandia, Uberlândia, Minas Gerais, Brazil,* ^d^ *Postgraduate Program in Epidemiology, Federal University of Pelotas, Pelotas, Rio Grande do Sul, Brazil,* ^e^ *Department of Dentistry, Faculdade São Leopoldo Mandic, Centro de Pesquisas São Leopoldo Mandic, Campinas, São Paulo, Brazil,* ^f^ *University of Saskatchewan, College of Dentistry, Saskatoon, SK, Canada,* ^g^ *Division of Preventive and Community Dentistry, School of Dentistry, Federal University of Uberlandia, Uberlândia, Minas Gerais, Brazil.*

**Keywords:** chloroquine, hydroxychloroquine, hyperpigmentation, oral mucosa

## Abstract

**Background:**

Chloroquine and hydroxychloroquine are 2 medications used to treat some systemic diseases.

**Objective:**

The aim of this scoping review was to assess the occurrence of oral pigmentation induced by chloroquine or hydroxychloroquine and to understand the pathogenic mechanism behind this phenomenon.

**Methods:**

The review was performed according to the list of PRISMA SrC recommendations and the JBI Manual for Evidence Synthesis for Scoping Reviews. MEDLINE (PubMed), Scopus, EMBASE, SciELO, Web of Science, Lilacs, and LIVIVO were primary sources, and “gray literature” was searched in OpenThesis and Open Access Thesis and Dissertations (OATD). Studies that screened the occurrence of oral pigmentation associated to the use of chloroquine or hydroxychloroquine were considered eligible. No restrictions of year and language of publication were applied. Study selection and data extraction were performed by 2 independent reviewers. The risk of bias was assessed through the JBI tool, depending on the design of the selected studies.

**Results:**

The initial search resulted in 2238 studies, of which 19 were eligible. Sixteen studies were case reports, 2 had case-control design and 1 was cross-sectional. Throughout the studies, 44 cases of oral pigmentation were reported. The hard palate was the anatomic region most affected with pigmentation (66%). According to the case reports, most of the lesions (44%) were bluish-gray. The minimum time from the beginning of treatment (chloroquine or hydroxychloroquine) to the occurrence of pigmentation was 6 months. The mean treatment time with the medications was 4.9 years, and the mean drug dosage was 244 mg. Most of the studies (63.1%) had low risk of bias (high methodological quality).

**Conclusions:**

The outcomes of this study suggest that hyperpigmentation depend on drug dosage and treatment length. Hyperpigmentation was detected after a long period of treatment with chloroquine or hydroxychloroquine.

## 1. Introduction

Chloroquine (CQ) and hydroxychloroquine (HCQ) are drugs originally designed to treat malaria.^[1]^ Over time, these drugs were included in treatment protocols for other infectious and immunological diseases.^[2,3]^ Adverse effects related to the use of CQ and HCQ have already been described, ranging from mild manifestations such as vomiting, diarrhea, and gastrointestinal disorders,^[4]^ to severe damage to the heart (ventricular hypertrophy, hypokinesia, heart failure, pulmonary arterial hypertension and valve dysfunction),^[5]^ skin, and mucosa (hyperpigmentation).^[4]^

Scientific evidence shows that, although hydroxychloroquine is generally well tolerated, dermatological adverse effects involving the skin, hair, or nails are a frequent and significant complication.^[6]^ As with skin, hair or nails, the ocular region can also be significantly altered, causing keratopathy, toxic retinopathy, and visual field defects.^[7,8]^ There are no review studies on oral mucosal hyperpigmentation with the use of CQ and HCQ, although it has been reported since 1964.^[9]^ The anatomical regions with the highest rates of occurrence are the palate,^[10]^ buccal and labial mucosa^[11]^ and tongue.^[12]^

This study aimed to carry out a scoping review of cases of reactive hyperpigmentation of the oral mucosa induced by CQ and HCQ. Understanding the pathogenic mechanism of mucosal hyperpigmentation was a secondary objective.

## 2. Methods

The scoping review protocol was performed according to Preferred Reporting Items for Systematic Review and Meta-Analysis Protocols (PRISMA-P).^[13]^ This review is reported following Preferred Reporting Items for Systematic reviews and Meta-Analyses extension for Scoping Reviews (PRISMA-ScR)^[14]^ and was conducted according to Joanna Briggs Institute Critical Appraisal tools in JBI Manual for Evidence Synthesis for Scoping Reviews.^[15]^ The Arksey and O’Malley methodological framework^[16]^ was employed to conduct this scoping review: identifying the research question, identifying relevant studies, study selection, data charting process, and summarizing and reporting results. All analyses were based on previous published studies; thus, no ethical approval and patient consent are required.

### 2.1. Research question

The eligibility criteria were based on the following research question: “Are chloroquine and hydroxychloroquine able to induce reactive hyperpigmentation of the oral mucosa as an adverse effect?”, where the “PCC” (Population, Concept, and Context) mnemonic was used to guide this scoping review, in which: P: General population using the medications (apart of sex, age or systemic disease), C: medication with CQ or HCQ, C: reactive hyperpigmentation of the oral mucosa.

### 2.2. Inclusion criteria

A study was eligible for inclusion if it reported primary data on the effect of chloroquine and hydroxychloroquine in inducing reactive hyperpigmentation of the oral mucosa in patients using the medications. As this is a scoping review, no language, publication date, or study design restrictions were applied. Thus, case reports, case series, observational studies (prospective and retrospective), randomized, and nonrandomized trials were included.

### 2.3. Exclusion criteria

The exclusion criteria were as follows: studies that involve only hyperpigmentation of the skin or the mucosa outside the oral cavity; and editorials and opinion articles.

### 2.4. Information sources, search, and selection of sources of evidence

The search was performed in June 2020 and updated in January 2022. MEDLINE (PubMed), Scopus, EMBASE, SciELO, Web of Science, Lilacs, and LIVIVO were primary sources, and “gray literature” was searched in OpenThesis and Open Access Thesis and Dissertations (OATD). Descriptors were selected using Medical Subject Headings (MeSH), Descriptors in Health Science (DeCS), and Embase Subject Headings (Emtree). Boolean operators (AND and OR) were used to combine descriptors and improve the search strategy by means of different combinations (Table 1). The search strategy for MEDLINE was adapted for the other databases, respecting their rules of syntax. The results obtained from the primary databases were initially exported to EndNote Web (Clarivate, Analytics, Philadelphia), excluding duplicates. The remaining references retrieved from “gray literature” were exported to Microsoft Word 2019 (Microsoft Ltd., Washington) software, and the duplicates were manually removed. The reviewers (PUJS and MBO) independently performed a methodical analysis of all study titles, specifically evaluating the study design and excluding studies that did not meet the inclusion criteria. Eligibility criteria were applied while reading abstracts from selected studies. The complete texts were read, and possible disagreements were taken to a third reviewer (LRP). In cases where the title or abstract provided insufficient information to accomplish a proper inclusion or exclusion decision, the full text was read to resolve any doubts. These strategies were performed to minimize the selection and publication bias.

**Table 1:** Electronic databases and applied search strategy.

**Database**	**Search strategy (January 2022)**
PubMed https://www.ncbi.nlm.nih.gov/pubmed	((“Hyperpigmentation” OR “Hypermelanosis” OR “Hypermelanoses” OR “Pigments, melanin” OR “Melanosis” OR “Melanoses” OR “Melanins”) AND (“Hydroxychloroquine” OR “Oxychlorochin” OR “Oxychloroquine” OR “Hydroxychlorochin” OR “Plaquenil” OR “Hydroxychloroquine Sulfate” OR “Chloroquine” OR “Chloroquine Sulphate” OR “Chlorochin”))
Scopus http://www.scopus.com/	((“Hyperpigmentation” OR “Hypermelanosis” OR “Hypermelanoses” OR “Pigments, melanin” OR “Melanosis” OR “Melanoses” OR “Melanins”) AND (“Hydroxychloroquine” OR “Oxychlorochin” OR “Oxychloroquine” OR “Hydroxychlorochin” OR “Plaquenil” OR “Hydroxychloroquine Sulfate” OR “Chloroquine” OR “Chloroquine Sulphate” OR “Chlorochin”))
Embase http://www.embase.com/	(‘hyperpigmentation’/exp OR ‘hyperpigmentation’ OR ‘hypermelanosis’/exp OR ‘hypermelanosis’ OR ‘hypermelanoses’ OR ‘pigments, melanin’ OR ‘melanosis’/exp OR ‘melanosis’ OR ‘melanoses’ OR ‘melanins’/exp OR ‘melanins’) AND (‘hydroxychloroquine’/exp OR ‘hydroxychloroquine’ OR ‘oxychlorochin’ OR ‘oxychloroquine’/exp OR ‘oxychloroquine’ OR ‘hydroxychlorochin’ OR ‘plaquenil’/exp OR ‘plaquenil’ OR ‘hydroxychloroquine sulfate’/exp OR ‘hydroxychloroquine sulfate’ OR ‘chloroquine’/exp OR ‘chloroquine’ OR ‘chloroquine sulphate’/exp OR ‘chloroquine sulphate’ OR ‘chlorochin’/exp OR ‘chlorochin’)
SciELO www.scielo.org	Hyperpigmentation OR Hypermelanosis OR Hypermelanoses OR Pigments, melanin OR Melanosis OR Melanoses OR Melanins AND Hydroxychloroquine OR Oxychlorochin OR Oxychloroquine OR Hydroxychlorochin OR Plaquenil OR Hydroxychloroquine Sulfate OR Chloroquine OR Chloroquine Sulphate OR Chlorochin
Web of Science http://apps.webofknowledge.com/	((“Hyperpigmentation” OR “Hypermelanosis” OR “Hypermelanoses” OR “Pigments, melanin” OR “Melanosis” OR “Melanoses” OR “Melanins”) AND (“Hydroxychloroquine” OR “Oxychlorochin” OR “Oxychloroquine” OR “Hydroxychlorochin” OR “Plaquenil” OR “Hydroxychloroquine Sulfate” OR “Chloroquine” OR “Chloroquine Sulphate” OR “Chlorochin”))
LILACS lilacs.bvsalud.org	((“Hyperpigmentation” OR “Hypermelanosis” OR “Hypermelanoses” OR “Pigments, melanin” OR “Melanosis” OR “Melanoses” OR “Melanins”) AND (“Hydroxychloroquine” OR “Oxychlorochin” OR “Oxychloroquine” OR “Hydroxychlorochin” OR “Plaquenil” OR “Hydroxychloroquine Sulfate” OR “Chloroquine” OR “Chloroquine Sulphate” OR “Chlorochin”))
LIVIVO https://www.livivo.de/app	((“Hyperpigmentation” OR “Hypermelanosis” OR “Hypermelanoses” OR “Pigments, melanin” OR “Melanosis” OR “Melanoses” OR “Melanins”) AND (“Hydroxychloroquine” OR “Oxychlorochin” OR “Oxychloroquine” OR “Hydroxychlorochin” OR “Plaquenil” OR “Hydroxychloroquine Sulfate” OR “Chloroquine” OR “Chloroquine Sulphate” OR “Chlorochin”))
OpenThesis http://www.openthesis.org/	(“Hydroxychloroquine” OR “Oxychlorochin” OR “Oxychloroquine” OR “Hydroxychlorochin” OR “Plaquenil” OR “Hydroxychloroquine Sulfate” OR “Chloroquine” OR “Chloroquine Sulphate” OR “Chlorochin”)
Open Access Theses and Dissertations (OATD) https://oatd.org/	((“Hyperpigmentation” OR “Hypermelanosis” OR “Hypermelanoses” OR “Pigments, melanin” OR “Melanosis” OR “Melanoses” OR “Melanins”) AND (“Hydroxychloroquine” OR “Oxychlorochin” OR “Oxychloroquine” OR “Hydroxychlorochin” OR “Plaquenil” OR “Hydroxychloroquine Sulfate” OR “Chloroquine” OR “Chloroquine Sulphate” OR “Chlorochin”))

### 2.5. Data charting process and data items

After study selection, reviewers performed a calibration exercise that consisted of selecting and extracting data from 3 randomly selected articles. The studies were analyzed independently by 2 reviewers (PUJS and MBO), and extracted data were further reviewed by another author (LRP). The following information was extracted: author, year of publication, study design, number of cases reported, sex, and associated disease. Also, the type of diagnostic examination and type of staining protocol (related to the pathogenic mechanism) were recorded. Information about the hyperpigmentation was extracted, namely the main color of the lesion, the anatomic region of occurrence, the medication, length of time using CQ or HCQ, drug dosage, and conclusion of the studies.

### 2.6. Critical appraisal of individual sources of evidence

After the process of study selection, risk of bias was assessed with the aid of the Joanna Brigs Institute critical appraisal checklist for case reports, case-control studies, and cross-sectional studies.^[17]^ The decision to apply each of the tools was based on the design of the selected studies, and was used to determine to what extent the study bias represented risks to the study design, setting, and analytical quality.

Two authors (PUJS and MBO) analyzed the studies with each tool and calculated the risk of bias. The studies were considered with high risk of bias when the percentage of positive answers to the questions in each tool was below 50%. Moderate risk of bias varied between 50% and 69%, and low risk of bias was considered when positive answers were ≥70%.t

### 2.7. Synthesis of results

The results of eligible studies were summarized in a descriptive/narrative manner, including study characteristics, and the therapeutic protocols, lesion features, and follow-up information about the hyperpigmented lesions of the cases reported. Study and case-specific characteristics were provided as absolute and relative frequencies. The descriptive analyses were performed using Stata 16.1 (StataCorp LLC, College Station, TX) software.

## 3. Results

### 3.1. Selection and characteristics of sources of evidence

Figure 1 shows the flowchart illustrates the search process, identification, inclusion and exclusion of eligible studies. During the first phase of study selection, 2238 results among 9 electronic databases, including the gray literature, were obtained. After the exclusion of duplicates, 1027 studies remained for title reading, out of which 831 were excluded because were not related to the research topic. Abstract reading was performed 196 times and resulted in 86 exclusions based on eligibility criteria. The remaining 110 full texts were read and led to 91 exclusions—mainly because the studies depicted hyperpigmentation on skin or on mucosa other than oral. Ultimately, 19 studies were included in the scoping review.

**Figure:**
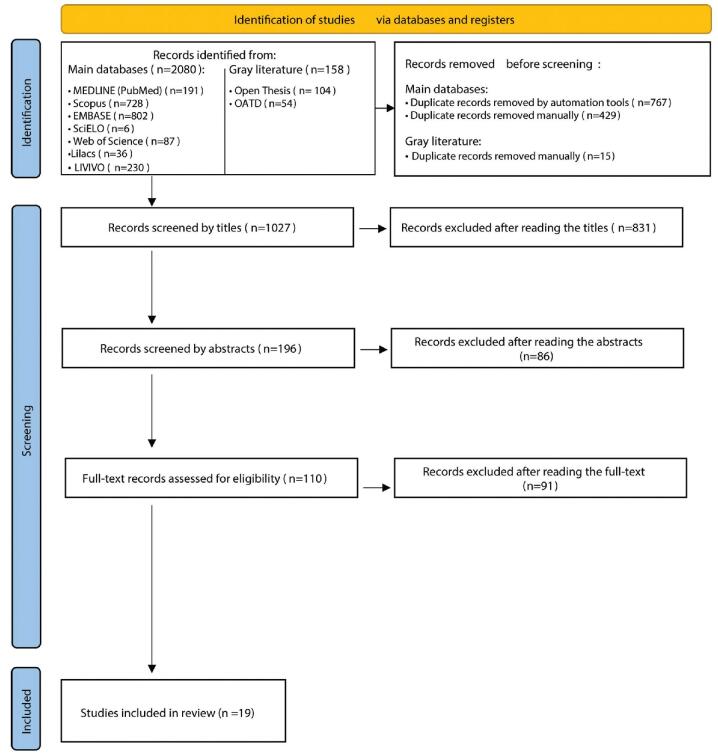
**Figure 1.** Flowchart of the search process.

The characteristics of the studies, including their design, medication evaluated, anatomic region of occurrence of hyperpigmentation, and main histopathological findings of the 19 eligible studies are reported in Table 2.^[9-12,18-32]^

**Table 2:** Summary of the main characteristics of the eligible studies.

**Author, year (ref)**	**Study design**	**Patients with oral pigmentation**	**Sex (n)**	**Medication (n)**	**Indication/overlying disease (n)**	**Anatomic region (n)**	**Diagnosis**	**Histopathological analysis**	**Histopathological features**	**Outcome (n)**
Piguet and Weissenbach, 1964^[9]^	Case report	1	Male	HCQ/CQ	Rheumatoid arthritis	Mucosa—hard palate, gum; skin—face	—	—	—	—
Langhof et al, 1965^[18]^	Case report	1	Male	CQ	Lupus erythematosus	Mucosa—hard palate; skin—leg	Biopsy	PAS; Perls’ reaction	Yellow and dark brown granules	Pigmentation has practically not decreased after discontinuing use of the drug. One y after exclusive therapy with acetylsalicyl acid, nicotinamide, and digoxin, the pigmentation on the palate and legs was practically faded
Levy, 1982^[19]^	Case reports	2	Male	CQ	Malaria	Mucosa—gum (2), lip (1); skin—back (1)	Puncture biopsy /biopsy	—	Increased melanin pigment in the basal layer and incontinence of pigment	The drug has been discontinued, no pigmentation reduction at 4 mo of follow-up (1)
Silveira et al, 2003^[20]^	Case reports	2	Female	CQ	Discoid lupus erythematosus	Mucosa—hard palate	Incisional biopsy /clinical examination	—	Pigments of exogenous origin in the form of dark granules dispersed in fibrous connective tissue subepitelial	The use of the medication was maintained without alteration in the form of pigmentation presentation (1)
Gallo et al, 2009^[21]^	Case reports	2	Female	CQ	Rheumatoid arthritis (1)/Sjogren syndrome (1)	Mucosa—hard palate (2); Skin—arm (1), leg (1)	Puncture biopsy /biopsy	Hematoxylin and eosin	Dark-brown granular pigment in the lamina propria, mainly located within fibroblasts, the subepitelial area and the perivascular region	—
Skare et al, 2011^[22]^	Case-control study	10	Male (1); female (9)	CQ	Rheumatoid arthritis (2)/systemic lupus erythematosus (8)	Mucosa—hard palate (2); cheek (7); diffuse (1)	—	—	—	—
de Melo Filho et al, 2012^[23]^	Case report	1	Female	CQ	Rheumatoid arthritis	Mucosa—hard palate, tongue, gum	Incisional biopsy	Hematoxylin and eosin;	Subepithelial deposition of tiny spherical brown-black structures scattered between collagen fibers and within macrophages	—
Kalampalikis et al, 2012^[24]^	Case report	1	Female	HCQ	Sjogren syndrome	Mucosa—gum	Biopsy	—	Basal hyperpigmentation without a significant increase of melanocytes as well as isolated melanophages in the lamina própria	The use of the medication was maintained
Martinelli-Kläy et al, 2012^[25]^	Case report	1	—	CQ	Malaria	Mucosa—hard palate	Biopsy	Perls’ reaction; Fontana-Masson stain	Small and médium-sized melanin granules in the macrophage cytoplasm; presence of ferric salts	Pigmentation persisted even after discontinuing the use of the medication—persistence for > 50 y after cessation of treatment
de Andrade et al, 2013^[10]^	Case reports	5	Female (3); male (2)	CQ (5)	Rheumatoid arthritis (5)	Mucosa—hard palate (5)	Incisional biopsy	Perls’ reaction; Fontana-Masson stain	Extra and intracellular darkbrown pigment in the connective tissue subjacent to the normal mucosa epithelium—hemosiderin	After 6 mo without chloroquine therapy, hyperpigmentation decreased significantly (1); The patient is being monitored (1); the drug has been discontinued (3)
Jallouli et al, 2013^[26]^	Case-control study	1	—	HCQ	Lupus erythematosus sistemic	Mucosa—hard palate	Biopasy	Hematoxylin and eosin; Fontana-Masson stain; Perls’ reaction	Pigmented granules throughout the dermis and hypodermis, interstitial macrophages and perivascular fibroblasts—melanin/iron stain	—
Takci et al, 2015^[27]^	Case report	1	Female	HCQ	Systemic lupus erythematosus	Mucosa—lip; skin—cheek, testa	Biopsy	Hematoxylin and eosin	Pigments with the presence of melanin in the basal keratinocytes	The drug has been discontinued. A decrease in pigmentation was seen in the first 6 mo, but there was no improvement
de Andrade et al, 2017^[28]^	Case report	1	Female	CQ	Rheumatoid arthritis	Mucosa—hard palate	Incisional biopsy	Perls’ reaction; Fontana-Masson stain; Immunohistochemistry with CD68	Subepithelial deposition of granular pigment mainly located between collagen fibers and within fibroblasts and macrophages—hemosiderin and melanin; macrophages containing intracellular pigment	The drug has been discontinued
Horta-Baas, 2018^[11]^	Case report	1	Female	CQ	Discoid lupus erythematosus	Mucosa—cheek, lip; nails	—	—	—	The medication was discontinued, and after 3 y of follow-up there was a decrease in nail pigmentation and persistence of hyperpigmentation in the mucosa
Manger et al, 2018^[29]^	Case report	1	Female	CQ	Sjogren syndrome	Mucosa—hard palate	Biopsy	Perls’ Prussian blue staining	Subepithelial granular pigment depositions among collagen fibers and within macrophages—hemosiderin	After discontinuation of treatment, hyperpigmentation regressed within a few months
Tosios et al, 2018^[30]^	Case report	1	Female	HCQ	Rheumatoid arthritis	Mucosa—hard palate, gum	Incisional Biopsy	Hematoxylin and eosin; Grocott [Gomori] methenamine-silver stain; Perls’ iron stain	Fine brown-yellow granules of approximately the same size in the reticular lamina propria—hemosiderin	The drug has been discontinued and pigmentation decreased considerably during the 30-month follow-up
Ladan and Shahid, 2019^[31]^	Case report	1	Female	HCQ	Rheumatoid arthritis	Mucosa—hard palate	Incisional biopsy	—	Normal stratification, maturation and deposition in the papillae of the connective tissue of melanin and hemosiderin	—
Chacón-Dulcey and López-Labady, 2020^[12]^	Cross-sectional	10	—	CQ (2) HCQ (8)	Lupus erythematosus (sistemic—6, cutaneous—4)	Mucosa—hard palate (8), cheek (2), tongue (1)	Incisional biopsy	Hematoxylin and eosin; PAS	Basal keratinocytes loaded with a brown pigment; histiocytes were observed in all cases near rounded brownish/black structures between the collagen fibers interpreted as antimalarial metabolites and in some cases mild inflammation with the presence of eosinophils	—
Godinho et al, 2020^[32]^	Case report	1	Female	CQ	Rheumatoid arthritis	Mucosa—hard palate	Incisional biopsy	—	Fragment of oral mucosa covered by parakeratinized stratified paved epithelium. On the lamina propria, in the subepithelial region and arranged in bands, exogenous black-colored pigments were noted, which sometimes were phagocytosed by macrophages	The medication was maintained according to the recommendations of the rheumatologist and hyperpigmentation showed no changes in its presentation
CD68 = cluster of differentiation 68; CQ = chloroquine; HCQ = hydroxychloroquine; PAS = periodic acid-Schiff; — = not reported.										

Most of the studies (84.2%) were case reports.^[9-11,18-21,23-25,27-32]^ Most of the case reports were conducted in Europe,^[9,18,24,25,27,29-31]^ whereas the cross-sectional and case-control studies were published in Europe^[26]^ and South America.^[12,22]^ Over 70% of the studies were published after 2011. The oldest eligible article, according to the year of publication, dates from 1964^[9]^ (Table 3).

**Table 3:** Description of the characteristics of the selected studies.

		**N**	**%**
Study design	
	Case report	16	84.2
	Case-control	2	10.5
	Cross-sectional	1	5.3
Study continent	
	Africa	1	5.3
	Europe	9	47.3
	North America	1	5.3
	South America	8	42.1
Year of publication	
	≤2000	3	15.8
	2001-2005	1	5.3
	2006-2010	1	5.3
	2011-2015	7	36.8
	2016-2020	7	36.8
n = absolute frequency; % = relative frequency.			

### 3.2. Critical appraisal within sources of evidence

The assessment of the risk of bias of the eligible studies is presented in Table 4. Among the 3 observational studies (crosssectional and case-control), all studies showed a low risk of bias.^[12,22,26]^

**Table 4:** Risk of bias assessed by the Joanna Briggs Institute Critical Appraisal Tools for use in JBI Critical Appraisal Checklist for analytical crosssectional studies, for case-control studies and for case reports.

**Authors (ref)**		**Q1**	**Q2**	**Q3**	**Q4**	**Q5**	**Q6**	**Q7**	**Q8**	**Q9**	**Q10**	**% Yes**	**Risk**
Analytical cross-sectional studies													
	Chacón-Dulcey and López-Labady^[12]^	✓	✓	✓	✓	✓	✓	✓	✓			100	Low
Case-control studies	
	Skare et al^[22]^	✓	✓	✓	—	✓	✓	✓	—	✓	✓	80	Low
	Jallouli et al^[26]^	✓	✓	✓	✓	✓	✓	✓	✓	✓	✓	100	Low
Case reports	
	Piguet and Weissenbach^[9]^	✓	—	✓	—	—	—	NA	✓			42.8	High
	Langhof et al^[18]^	✓	✓	✓	✓	✓	✓	NA	✓			100	Low
	Levy^[19]^	✓	—	✓	✓	U	U	NA	✓			57.1	Moderate
	Silveira et al^[20]^	✓	U	✓	U	U	—	NA	✓			42.8	High
	Gallo et al^[21]^	✓	—	✓	✓	—	—	NA	✓			57.1	Moderate
	de Melo Filho et al^[23]^	✓	✓	✓	✓	—	—	NA	✓			71.4	Low
	Kalampalikis et al^[24]^	✓	✓	✓	✓	U	—	NA	✓			71.4	Low
	Martinelli-Kläy et al^[25]^	U	✓	✓	✓	✓	✓	NA	✓			85.7	Low
	de Andrade et al^[10]^	✓	U	✓	✓	✓	U	NA	✓			71.4	Low
	Takci et al^[27]^	✓	✓	✓	✓	✓	✓	NA	✓			100	Low
	de Andrade et al^[28]^	✓	✓	✓	✓	✓	—	NA	✓			85.7	Low
	Horta-Baas [11]	✓	✓	✓	—	✓	✓	NA	✓			85.7	Low
	Manger et al^[29]^	U	—	✓	✓	✓	U	NA	✓			57.1	Moderate
	Tosios et al^[30]^	✓	—	✓	✓	✓	✓	NA	✓			85.7	Low
	Ladan and Shahid^[31]^	✓	—	✓	✓	—	—	NA	✓			57.1	Moderate
	Godinho et al^[32]^	✓	✓	✓	✓	✓	✓	NA	✓			100	Low
✓ = yes, — = no, NA = not applicable, U—unclear.													

In case report studies, the only question of the JBI tool not applicable was Q7 because the hyperpigmentation itself was the analyzed adverse effect within the studies.^[9-11,18-21,23-25,27-32]^ Q1 was not clear in 2 studies^[25,29]^ because the authors did not report on the sex and medical history of the patients. The lack of detailed medical history also hampered higher scores in Q2.^[9,10,20,21,29-31]^ Positive answers were detected for the studies in Q3 and Q8 (questions related to clinical conditions), but for Q4, 2 studies^[9,11]^ had negative score because of lacking histopathological testing for the diagnosis of hyperpigmentation. For the same question, another study lacked clarity because the authors reported histopathological findings only for half of the cases.^[20]^ In Q5, the treatment protocols were not clearly defined, or were defined only for half of the participants.^[19,20,24]^ Four studies did not treat (intervention) patients with hyperpigmentation.^[9,21,23,31]^ Q6 was considered unclear when the postinterventional condition was described for the total sample, and not individually per partcipants,^[10,19]^ or when the time frame of drug suspension was not reported.^[29]^ In 7 studies,^[9,20,21,23,24,28,31]^ no interventional post-clinical condition was mentioned.

### 3.3. Results of individual sources of evidence

Together, the case reports^[9-11,18-21,23-25,27-32]^ described the situation of 23 patients. Most cases were from female individuals, with mean age of 55.3 years. Over 50% of the patients were using CQ or HCQ for the treatment of lupus, followed by rheumatoid arthritis, malaria, and Sjögren syndrome. The mean treatment length with CQ and HCQ was 4.9 years (Table 5). As depicted in Figure 2, the most frequent hyperpigmentation in the case report studies were bluish-gray or dark gray. The hard palate was the anatomic region most affected by hyperpigmentation (65.9%), followed by the buccal mucosa (22.7%).

**Table 5:** Characteristics of the cases described by the selected case report studies.

		**N**	**%**
Sex^*^	
	Female	16	72.7
	Male	6	27.3
Disease treated	
	Rheumatoid arthritis	12	52.3
	Lupus	5	21.7
	Malaria	3	13.0
	Sjögren syndrome	3	13.0
		Mean	SD
Age, y		55.3	3.09
Length of HCQ use, y		4.9	0.87
CQ/HCQ dosage, mg		244	17.12
Case-control and cross-sectional studies were not described since demographic characteristics specifically for cases of oral hyperpigmentation were not available.			

^*^ Characteristic not reported by one of the selected case report studies.

**Figure:**
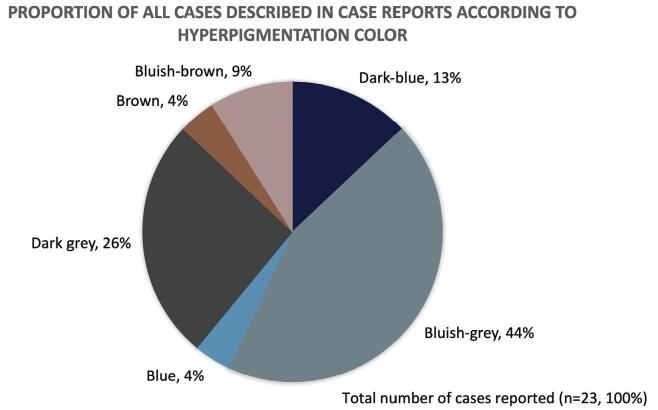
**Figure 2.** Proportion of all cases described in case report studies according to their hyperpigmentation color.

The single cross-sectional study selected^[12]^ revealed that 10% of the patients under use of CQ or HCQ had multiple hyperpigmented stains on the oral mucosa. Most of these patients (80%) were using HCQ. In case-control studies,^[22,26]^ the number of patients with mucosal hyperpigmentation was low: in the study by Jallouli et al,^[26]^ of the 24 patients in the case group (who had some hyperpigmentation caused by the use of HCQ), only 1 patient had hyperpigmentation of the palate mucosa; in the study by Skare et al,^[22]^ of the 209 patients who used HCQ or CQ (case group), 159 patients had some cutaneous side effects, of which only 10 patients had hyperpigmentation on the cheek or hard palate mucosa after using CQ (mean time of usage of 60.5 months). Besides, the authors found a statistical association (*P* = .0018) between mucous hyperpigmentation and antimalarial drug (CQ and HCQ).^[22]^

## 4. Discussion

CQ and HCQ are drugs associated with multiple intracellular processes, especially with antimalarial, anti-inflammatory, and immune responses.^[33]^ The specificity of CQ against malaria consists of an active mechanism of accumulating acidic vesicles, lysosomes, and Golgi complex leading to enzyme dysfunction (depending on the gradient of hydrogen ions in the medium.^[34,35]^ In patients with lupus, CQ acts on pro-inflammatory cytokines—reducing interleukin 6, 18, and tumor necrosis factor alpha.^[36]^

For other diseases, such as malaria and lupus, CQ, and HCQ proved their effectiveness and are well-tolerated drugs under therapeutic doses.^[33-42]^ However, they involve some adverse effects. The most common are nausea, diarrhea, abdominal discomfort, skin eruption, and alopecia.^[43]^ Both drugs derivate from quinine and share the same mechanism of physiopathology toxicity.^[43]^ Acute intoxication with CQ and HCQ may include depression of the respiratory system and central nervous system, and seizure.^[44]^ Cardiovascular toxicity is reported as well, especially from the blockage of sodium and potassium channels.^[45]^ The cumulative dosage—>100 g, may lead to neurosensory deafness, visual deficit, corneal opacities, and irreversible retinopathy.^[33]^ Agranulocytosis, aplastic anemia, hypersensitivity reactions, hepatitis, myopathy, neuropathy, and cardiomyopathy have been reported with the chronic use of CQ and HCQ.^[46]^

Pigmentation induced by CQ and HCQ also figures among adverse effects described in the scientific literature. The estimated incidence of pigmentation is 7.3%.^[26]^ Studies with the cultivation of pigmented cells revealed that CQ has an extensive adherence and retention to melanin.^[47,48]^ The influence of the drug on pigmentation, however, depends on drug dosage and length of time that the drug is used.^[47]^ The mechanism of formation of the CQ-melanin complex involves strong bridging of hydrophobic interaction and electrostatic attraction between the protonated ring system of the CQ and the ortho-semiquinone group of the melanin. In specific sites of weak reaction, might be observed ionic bonds between the aliphatic nitrogen of the chloroquine molecule and the carboxyl groups of melanin.^[49]^ Weak bonding also includes Van der Waals forces in the aromatic rings of CQ and in the aromatic indolic nuclei of the melanin.^[49,50]^

The pigmentation of the oral mucosa associated with quininederived drugs was firstly reported in 1945 by Lippard and Kaeur.^[51]^ The authors described the staining as predominantly bluish/brownish.^[51]^ Similarly, the present systematic review found predominance of bluish-grey staining on the hard palate within the eligible articles. The predominance of staining on the palate is explainable because the mucosal tissue of the palate is thin and closely adjacent to the underlying bone. This condition might allow the superficial deposition of the drug metabolites.^[52]^

The diagnosis of drug-induced hyperpigmentation is based on medical history and clinical signs. Biopsy, however, may be useful to confirm the origin of the pigment.^[53]^ Only 5 cases included in this systematic literature review did not report diagnosis via biopsy procedures and/or clinical assessment. Atypical cases of hyperpigmentation may rely on the biopsy to distinguish druginduced staining from oral melanoma—which may show hyperpigmentation as the only clinical sign in early stages.^[54]^

According to De Andrade et al,^[10]^ oral pigmentation associated to antimalarial drugs is reversible after drug discontinuation or reduction of drug dosage. It must be noted, however, that information on the reversibility of oral pigmentation induced by medication (CQ and HCQ) is still scarce. To guarantee the best practices and to avoid worse prognoses, patients with oral pigmentation related to the use of CQ and HCQ must be referred to ophthalmologists for the assessment of retinopathy.^[10]^ Despite the safe recommendation found in the scientific literature, there is no study addressing the potential association of oral pigmentation and other systemic adverse effects of the CQ and HCQ. For this reason, oral pigmentation must not be used as a marker of drug toxicity.

The outcomes of the eligible studies varied from lesion (staining) disappearance, reduction, and persistence. It is important to note that not only the clinical aspect of the lesion varied, but also the therapeutic management of patients, namely the maintenance of the drug, discontinuation or replacement/association with another drug. Considering the questionable reversibility of the condition, clinical follow-up becomes fundamental to understand the behavior of the drug-induced pigmentation. This study presents some limitations, including the limited number of studies, in addition to the focus on a single adverse effect (hyperpigmentation of the oral mucosa). Additionally, most studies included in this review were case reports, hence we were not able to calculate a combined effect of CQ and HCQ use on hyperpigmentation of the oral mucosa as this would be a biased estimate. Furthermore, the inclusion of studies with low strength of evidence and the lack of experimental studies on the adverse effects of CQ and HCQ highlight the need for further studies on the subject.

## 5. Conclusions

The outcomes of this study suggest that hyperpigmentation depend on drug dosage and treatment length. Hyperpigmentation was detected after a long period of treatment with CQ or HCQ. This study focused on a single adverse effect that could be related to the use of CQ and HCQ; however, patients affected by hyperpigmentation are recommended to be referred to ophthalmologists and cardiologists for a multidisciplinary checkup.

## Author contributions

PUJS, MBO, and LRP conceived the idea and had full roles in the identification, article review, data extraction, quality assessment, analysis, draft writing and revision of the manuscript. WAV, SVC, CB, AF and WLS took major roles in the analysis, manuscript draft preparation and revision. All authors read and approved the final version of the manuscript to be considered for publication. All authors also agreed to be equally accountable for all aspects of this research work.

**Conceptualization:** Pedro Urquiza Jayme Silva, Ademir Franco, Luiz Paranhos.

**Data curation:** Pedro Urquiza Jayme Silva, Millena Barroso Oliveira.

**Formal analysis:** Pedro Urquiza Jayme Silva, Millena Barroso Oliveira, Sérgio Vitorino Cardoso, Cauane Blumenberg.

**Funding acquisition:** Luiz Paranhos.

**Investigation:** Millena Barroso Oliveira.

**Methodology:** Pedro Urquiza Jayme Silva, Millena Barroso Oliveira, Walbert Vieira, Sérgio Vitorino Cardoso, Cauane Blumenberg, Walter Luiz Siqueira.

**Supervision:** Walter Luiz Siqueira.

**Validation:** Walter Luiz Siqueira.

**Visualization:** Pedro Urquiza Jayme Silva, Millena Barroso Oliveira, Walbert Vieira, Sérgio Vitorino Cardoso, Cauane Blumenberg, Ademir Franco, Walter Luiz Siqueira, Luiz Paranhos.

**Writing - original draft:** Pedro Urquiza Jayme Silva, Millena Barroso Oliveira, Walbert Vieira, Sérgio Vitorino Cardoso, Cauane Blumenberg, Ademir Franco, Walter Luiz Siqueira, Luiz Paranhos.

**Writing - review & editing:** Pedro Urquiza Jayme Silva, Millena Barroso Oliveira, Walbert Vieira, Sérgio Vitorino Cardoso, Cauane Blumenberg, Ademir Franco, Walter Luiz Siqueira, Luiz Paranhos.
